# Matching Mobile Crisis Models to Communities: An Example from Northwestern Ontario

**DOI:** 10.1007/s11414-024-09882-7

**Published:** 2024-04-30

**Authors:** Jillian Zitars, Deborah Scharf

**Affiliations:** Department of Psychology, Lakehead University, 955 Oliver Rd, Thunder Bay, ON P7B 5E1 Canada

**Keywords:** Mobile crisis response team, Program evaluation, Crisis response models, Rural and northern contexts

## Abstract

Police are often the first to encounter individuals when they are experiencing a mental health crisis. Other professionals with different skill sets, however, may be needed to optimize crisis response. Increasingly, police and mental health agencies are creating co-responder teams (CRTs) in which police and mental health professionals co-respond to crisis calls. While past evaluations of CRTs have shown promising results (e.g. hospital diversions; cost-effectiveness), most studies occurred in larger urban contexts. How CRTs function in smaller jurisdictions, with fewer complementary resources and other unique contextual features, is unknown. This paper describes the evaluation of a CRT operating in a geographically isolated and northern mid-sized city in Ontario, Canada. Data from program documents, interviews with frontline and leadership staff, and ride-along site visits were analyzed according to an extended Donabedian framework. Through thematic analysis, 12 themes and 11 subthemes emerged. Overall, data showed that the program was generally operating and supporting the community as intended through crisis de-escalation and improved quality of care, but it illuminated potential areas for improvement, including complementary community-based services. Data suggested specific structures and processes of the embedded CRT model for optimal function in a northern context, and it demonstrated the transferability of the CRT model beyond large urban centres. This research has implications for how communities can make informed choices about what crisis models are best for them based on their resources and context, thus potentially improving crisis response and alleviating strain on emergency departments and systems.

**Supplementary Information:**

The online version contains supplementary material available at 10.1007/s11414-024-09882-7.

## Introduction

Due to the nature of many emergency response systems, police are often the first to encounter people living with mental illness when they are in crisis (i.e., a situation in which an individual is under extreme emotional or psychological distress and may be at risk of harming themselves or others).^1,2^ This is problematic because mental health response is not the primary mandate of police agencies and other professionals are better trained to address community members’ mental health needs.^1,2^ To improve crisis services and to keep communities safe, police have partnered with mental health agencies to employ various collaborative crisis response models. The purpose of this study is to present the structures, processes, and outcomes of a joint police-mental health crisis response service operating in a geographically isolated and northern mid-sized city, which unlike larger urban contexts, have not been well-described.

In Canada, co-responder teams (CRTs) are emerging as the predominant approach to improving police response to mental health crisis calls.^3,4^ This approach is less prevalent in the USA, where crisis intervention training (CIT; i.e., enhanced mental health-specific officer training, strong partnerships with community-based services, police-only response) is more common.^5^ However, the CRT model is growing in popularity in the USA, is in alignment with the Substance Abuse and Mental Health Services Administration’s^6^ best practice guidelines for behavioral health crisis response, and is part of a comprehensive crisis response system that is needed to complement the recently established National Suicide Prevention Lifeline 988.^7^ Lessons learned from studies on CRTs in Canada can inform successful implementation of CRT models in other countries including the USA.

The CRT model differs from the CIT model as it uses a co-response approach, where police and mental health professionals respond together to crisis calls. CRTs are intended to deescalate situations involving people living with mental illness through immediate assessment, support, and intervention while simultaneously improving care quality and reducing system costs through reduced use of force, increased emergency department (ED) and jail diversion, and improved connections to the less restrictive suitable services in the community.^8^ CRTs may also support quality of care and system goals through related service actions, such as following up with clients after a mental health crisis to reduce the need for future emergency service use and enabling individuals to remain in their usual environment with their natural supports when appropriate.^9–11^

Previous studies have shown that CRTs reduce involuntary hospital transports, ED presentations, and wait times, and increase referrals to community-based services compared to traditional police response.^12,13^ This research, however, largely comes from CRTs in large urban settings (see review by Ghelani et al.^13^; all but one study occurred in urban contexts). How CRTs operate in rural, Northern, or remote settings with different populations, cultural issues, resources, and geography is largely unknown.

### Crisis Response in Smaller Communities

Crisis response teams in rural or isolated areas may have different resources from those in large urban centres, which can impact how CRTs are implemented and sustained.^14^ Reduced funding, workforce, and complementary community-based resources are challenges to implementing CRTs in small communities.^9^ CRTs operating in rural settings may also serve a larger geographical area and thus take longer to travel to calls for service.^15,16^

To our knowledge, the only available study of a CRT operating in a rural and suburban context^15^ found that CRT services were superior to general police patrol for reducing time spent on crisis-related calls, costs, and involuntary apprehensions, while also increasing referrals to community resources from pre- to post-implementation. While this single study suggests that CRTs can be successfully adapted for different contexts, more evidence is needed to support the implementation of CRTs in varied settings. Not all smaller and rural communities are alike, and further examination of community characteristics, such as community resources, population, and geographical features, is needed to inform the implementation of CRTs in less populous, rural, and northern areas.

### Aligning CRT Approaches with Contexts

Current research on CRTs is also limited insofar as it is heavily outcome focused. Outcome-focused studies that do not simultaneously examine program context, structures, and processes run the risk of the following: (1) drawing conclusions about the entirety of a model (i.e., effective or not effective) without explicating and assessing the mechanisms of actions that could be impacting model effectiveness, and (2) obfuscating opportunities for program improvement. An extended Donabedian framework^17–20^ is a commonly used framework for quality assessment of healthcare delivery. The extended Donabedian framework assesses healthcare quality by examining context (e.g., regional context, demography, geography), structure (e.g., resources, organizational features, policies), process (e.g., activities being carried out, the extent to which a program operates as intended), and outcomes (e.g., client dispositions, resource-savings). This approach is also aligned with the emergent literature calling for a focus on crisis response *systems* over crisis response services (i.e., coordinated crisis services that are suited for differing degrees of crisis severity, including somewhere that individuals can be diverted to from the hospital and that can deliver post-crisis care).^11,21,22^ The extended Donabedian framework can be used to explicate where CRTs fit within other community services (including crisis systems) and, therefore, can also help explain *how* and *why* a model thrived or failed to succeed in a given context.

### The Current Study

The current study uses an extended Donabedian framework to examine factors that influence CRT function in a geographically isolated and northern mid-sized city in Ontario, Canada. The city where this study occurred has a population of approximately 110,000 and land area of approximately 208 mi,^2^ and it serves as a hub providing most health, education, and social services for Northwestern Ontario. The CRT in this community is a co-located, or “live-embedded” CRT model.^23^ It was launched on January 1, 2021, and was funded by a provincial grant to the city police force and a mental health agency. The CRT operates 24/7 with the goal of at least one police-crisis worker team on the road at all times. This CRT uses police vehicles to respond to mental health crisis calls identified by police dispatch and the crisis line. Officers self-elected to receive training (training detailed in “Results”).

The current CRT was developed following a 2-year trial period of a previous mobile crisis response team approach in which a two crisis worker-team was dispatched to police mental health calls for assistance. While the previous model was found to be highly successful, it was also limited by its hours of operation (2 pm – 2 am) and delays from when the police first responded to the call and the time that the crisis workers arrived on the scene. In response to these challenges, program leadership designed the current CRT to fill these gaps and pilot the co-responder model in the community.

## Methods

Data collection for this case study consisted of semi-structured interviews, program document review, and ride-along site visits. Partner agencies were consulted throughout the research design process. This study was reviewed and approved by Lakehead University’s Rearch Ethics Board (#1469198).

### Interview Participants

A total of* N* = 13 individuals participated in study interviews. Two administrators were recruited from each partner agency (mental health agency, police agency, and hospital;* n* = 6). The study details and invitation were distributed to leadership at the partner organizations, who shared it with frontline staff multiple times. Using convenience sampling, three frontline staff were recruited from both the mental health agency and police agency, and one from the hospital for a total of *n* = 7 frontline staff interviews. The sample includes nearly half of the mental health, and approximately one-quarter of the police CRT workforce at any given time, although the size of the workforce at each agency changed throughout the study. Staff were not incentivized to take part, and participation was voluntary.

### Data Sources

#### Interviews

Interviews were conducted by JZ and followed semi-structured interview guides for police frontline staff, crisis workers, hospital staff, and leadership across agencies (see Supplemental Documents). The interview guides were developed by JZ and DS and were reviewed and approved by leadership at the mental health and police agencies. Guided by the extended Donabedian framework, the interview questions addressed the structure (e.g., resources or policies), process (e.g., typical operations), and outcomes (e.g., referrals) of the CRT, and program contextual factors.

#### Program and Agency Documents

Program and agency documents included crisis intervention training (CIT)^5^ curriculum, job postings, progress reports (including program statistics for number of calls attended and client outcomes (e.g., Zitars and Scharf^24^), and a quality improvement focus group report commissioned by the mental health agency and previously completed by the research team.

#### Ride-along Site Visits

We conducted ride-along site visits with the program to contextualize our data and confirm our findings. Observational data was recorded in site visit matrices.

### Procedures

All interviews were conducted over Zoom and lasted an average of 55 min (*SD* = 20.83). Audio recordings were transcribed using Otter.ai transcription software (version 1), and then reviewed and corrected for accuracy. Program and agency documents were collected from partner agencies. These documents included weekly service statistics, a job posting for the crisis worker position, and annual reports to funders and community. Ride-along site visits occurred three times (one day shift; two night shifts) and were each approximately 4 h in length. When safe and appropriate to do so, JZ observed and queried activities and processes.

### Data Analysis

Audio transcriptions were coded in the ATLAS.ti software (version 23). Braun and Clarke’s^25,26^ framework for thematic analysis was used to systematically identify the themes that arose from the interviews. This analysis was both deductive (i.e., using an extended Donabedian framework^18,20^ to inform possible themes and/or important aspects of the program) and inductive (i.e., using the data to determine the themes without preconceptions or theories). JZ primarily conducted the thematic analysis and DS reviewed transcripts, reviewed and revised the themes for clarity, and ultimately endorsed the final results. We used a two-step qualitative approach to determining data saturation (i.e., initially demonstrated when no new details and/or perspectives were arising in the interviews; confirmed in the data coding process when new codes/themes were not generated). Document and site visit data were incorporated into the analysis for validation and to identify additional program components not emerging from the other data sources.

## Results

We identified 12 themes and 11 subthemes across the four domains of the Donabedian framework. Table 1 presents an overview of the themes, subthemes, and their definitions. Supporting quotes are referenced throughout and are in Table 2.

**Table 1:** Qualitative Analysis Themes

Theme	*Subtheme*	Description	Supporting quote #s
Context			
Meeting community needs		The CRT model meets community needs by addressing the high proportion of social service calls, tailoring training towards the needs of the community, and by using an approach that is better suited for community demographics	1
The northern context		Contextual factors of Northern Ontario, including its geographic isolation, extreme temperatures, workforce issues, and how police mandate may vary in these contexts	2
Complementary community resources		Services available that compliment a program that relies upon providing low-barrier referrals to operate	N/A
	* A fair amount*	A fair variety of different community-based services in the city	N/A
	* Resource accessibility*	The relative accessibility of existing community-based resources	3; 4; 5; 6;
	* Resource gaps*	Gaps in the different areas of services available, including long-term services	N/A
	* Crisis-specific services*	Different crisis-specific service options are critical to the operation of the CRT	7
Structure			
Training		Different trainings that contribute to the knowledge base of CRT frontline staff for them to fulfill their role	8
Equipment/uniforms		The equipment and different uniforms necessary for CRT frontline employees	9
Space		The co-located office space for crisis workers	N/A
Staffing		How the program is staffed for each role	N/A
	* Staffing shortages*	Shortages in the number of crisis worker staff and number of CRT officers	N/A
	* Staff fit*	The relative fit between the employee and the frontline RT staff, including past experience and personality or character traits	10
Process			
The dispatch process		The overall process of dispatching the CRT to calls	N/A
	* Responding to non-mental health calls*	The CRT responding to calls for service that are not mental health-related	11
	* Call volume and triage*	The high mental health call volume and how the team is triaged to these calls	12
Call resolution		The process of resolving a call for service	N/A
	* Diversion, crisis de-escalation and referrals*	Diverting individuals from the hospital or jail by de-escalating crisis situations and providing referrals to community-based services	N/A
	* Hospital transfers*	Transfers to the hospital, either under the Mental Health Act or voluntary	13
	* Nowhere else to go*	The lack of suitable places for someone experiencing crisis other than the hospital at times	14
Outcomes			
Client Outcomes		The different outcomes of clients	15;16
Quality of Care		The quality of care provided by the CRT	17
Program efficiencies		The general savings of resources through the CRT program, including police, hospital, and community savings	18

**Table 2:** Interview Quotes

Quote #	Domain	Theme (*and subtheme if applicable*)	Quote
1	Context	Meeting community needs	“80% of our calls are social service and crisis-related, and [so the CRT] provides a good service to the community in responding to mental health issues, versus just a police response.” (Police Leadership)
2	Context	The northern context	“We’re dealing with big city social issues in a small town with small budgets, you know, so that I think part of it too, is that for, like, I know that in other centres, like I have officers, friends from bigger centres, like [name of urban centre], right? They don’t respond to things like this … So, [the CRT] is the perfect fit for what’s happening here in the North.” (Crisis Worker)
3	Context	Complementary community resources (*Resource accessibility*)	“That’s one of the challenges with doing [the CRT], like any 24-h program, somebody can be super well supported in the community and have all these resources, but it’s 3 am on a Saturday night and you’ve got to look at all the circumstances. Is this person able to wait until those resources are available? If not, we might still be having to make decisions based on that person’s safety or other people’s safety in the moment.” (Crisis Worker)
4	Context	Complementary community resources (*Resource accessibility*)	“We found out that a program, all of us had left verbal voicemails for thinking that was a sufficient referral. None of us ever heard back from them saying that is not sufficient. But when [the crisis office] called to follow up to make sure they got the referral [during business hours] so that they can actually talk to somebody. Their response was just ‘Oh, we don’t take referrals by phone.’” (Crisis Worker)
5	Context	Complementary community resources (*Resource accessibility*)	“I think we have the [programs/services], it’s just if there’s the resources are staffed [thus, able to accept clients optimally]… I think that [our community] is very creative and innovative, of how to meet the needs of the community, by stretching their mandates and working in the gray to come together. I think a lot of it is you might be given [funds to run a program]. But how do you run the program if you don’t have people to?” (Mental Health Agency Leadership)
6	Context	Complementary community resources (*Resource accessibility*)	“Especially with waitlists in city too. Waitlists for everything. For psychiatry, psychology, children’s mental health [services], treatment for addictions, like there’s a waitlist for everything, right? So everybody is just… some people are just teetering, and there’s going to be a tipping point if you wait long enough, right?” (Hospital Leadership)
7	Context	Complementary community resources (*Crisis-specific services*)	“It’s like that continuum, right? Because not everyone in crisis goes right to an [CRT crisis severity] level, and it’s that assessment of [their needs].” (Mental Health Agency Leadership)
8	Structure	Training	“So, [the policer officers] get some mental health training there that we do through their career, they get reintroduced to it in different ways. But the CIT training … really allows [officers] to think of other strategies that may have been not due to potentially training capacity issues, in the amount of time that things have to learn as a new recruit… The CIT training really allows them that dedicated time to work on [mental health-related skills] and see different perspectives as a result of taking that training with crisis workers. But it’s very focused on those strategies they would utilize in real calls.” (Police Leadership)
9	Structure	Equipment/uniforms	“[The mental health agency is] making efforts and I appreciate that. But the officers get an annual budget of a set amount of money for both a winter and a summer pair of shoes. And they get that every single year [compared to a one-time voucher]. So, we’re working in the same circumstances, often without all of the resources.” (Crisis Worker)
10	Structure	Staff fit	“I know lots of people really want to do [the CRT] because they think it’s so cool. But because [new crisis worker recruits] are ill-equipped... Because it’s not the feel-good provider, right? Like people just want us to make people feel good. That’s not what we do... I think there’s just a little bit of a lack of understanding with what we do. And therefore [there is a lack of understanding of] what a good candidate looks like, and how to follow up with a good candidate.” (Crisis Worker)
11	Process	The dispatch process (*Responding to non-mental health calls*)	“We’re stuck on a call that’s not mental health, it’s definitely police, but we can’t leave to attend to what is now a mental health call or a call where we can be of use.” (Crisis Worker)
12	Process	The dispatch process (*Responding to non-mental health calls*)	“There’s other days where there’s five or six calls for mental health concerns on the screen, and we have to triage what’s the most important one to go to.” (Police Officer)
13	Process	Call resolution (*Hospital transfers*)	“We’ve had times where we’re sitting in the ambulance bay for hour upon hour upon hour with people who, on the initial assessment of bringing them in, really needed to attend hospital. Seven hours later of waiting in an ambulance bay. Guess what? Their presentation may have changed [to less severe].” (Crisis Worker)
14	Process	Call resolution (*Nowhere else to go*)	“Yeah, there is no in-between. It’s hospital or they stay in the street, which doesn’t happen… But it usually ends up hospital and then you sit there for six hours. And then the next time something happens, people are like, ‘I am not going to the hospital.’” (Crisis Worker)
15	Process	Call resolution (*Nowhere else to go*)	“[Talking about a specific incident on ride-along] Weeks prior, if I was the [Mental Health Assessment Team] nurse or manager getting this person admitting admitted to my unit. Looking at the facts around it, it would have been like, "Okay, well, why were they admitted this wasn’t necessarily appropriate intervention here", right? But having seen it from their side of things, really understanding that they’re working with what they have, and that the ultimate goal is to keep the person safe. Recognizing that, going to the cell within the police station for the night wasn’t appropriate. Detox wasn’t appropriate. And so, where else do we have? It kind of made me realize that there are some gaps [in services] out there where, you know, an individual has nowhere else to go except for our emergency department.” (Hospital Leadership)
16	Outcomes	Client outcomes	“So we’re definitely meeting those like, I think we have really high hospital diversion numbers… We have to be resourceful sometimes, right? So like asking the types of questions like finding safe places, finding safe people, you know, but I feel like we’re definitely we’re exceeding our outcomes, based on what I can see from the numbers.” (Crisis Worker)
17	Outcomes	Quality of care	“Family [members] are also supported through [the CRT] as well. Sometimes mental health patients don’t quite understand that they’re unwell. And it’s their families that’s advocating for their care. So [the CRT] is really able to validate the family members, and support them and getting them to hospital.” (Hospital Worker)
18	Outcomes	Program efficiencies	“We don’t get to see how many patients that they prevent from coming to hospital. But with my involvement with crisis response through doing training with [the CRT] and police… We’ve heard the stories about [the CRT], so I know that they’re preventing a lot more than are coming to hospital.” (Hospital Staff)

### Demographics

The mean age of participants was 44.31 (*SD* = 10.55; range: 27–60). Ten (76.92%) participants identified as female and three (23.08%) identified as male. Job titles included the following: Constable, staff sergeant, crisis worker, manager, supervisor, registered nurse, and charge nurse. All participants had some post-secondary education. All frontline police and crisis worker staff reported receiving specialized CRT training.

### Context

All participants reported that they believed the CRT model was suitable for the community context. Within this domain, three themes of *meeting community needs*, *the northern context*, and *complementary community resources* emerged.

#### Meeting Community Needs

Participants reported that the CRT model was suitable because it met the community’s need for improved mental health and addiction services, housing, solutions for crime and violence, and services tailored for youth. Overall, all police leadership, officers, and crisis workers noted the high proportion of social-service calls received by the police agency, making the CRT model well-suited to the community’s needs (Quote #1).

Participants highlighted the city’s diverse population, namely the large Indigenous population, and the complicated history and built distrust between Indigenous individuals and the police agency. Respondents indicated that the CRT provided a much-needed softer-facing approach to crisis care. While participants did not state that CRT services were extensively culturally responsive, participants endorsed that the CRT was an improvement from traditional police-only response.

#### The Northern Context

Participants described how the community’s geographic isolation and its status as a hub for Northwestern Ontario resulted in few other resources nearby, low or no workforce in neighbouring communities to draw from, and many individuals from other northern and more remote communities coming to this city for services, further straining local resources, and creating gaps in knowledge about many of the individuals that the team serves (e.g., health history was unlikely to be in accessible databases). Moreover, the city’s extremely cold temperatures were reported to have affected staff equipment needs (e.g., warm coats, boots) on the road and during hospital transfers.

Lastly, the northern context was identified as affecting the types of calls that the police respond to (i.e., police mandate) including many social service calls that do not fit under the traditional police safety mandate (e.g., mental health and addictions calls, non-criminal calls for service) because no other appropriate or immediate services (e.g., crisis respite beds, detoxification centres) were available (Quote #2).

#### Complementary Community Resources

Largely, participants expressed that there were enough services to enable the CRT to run, but not enough for it to function optimally. Four sub-themes arose pertaining to the strengths and limitations of community services: *A fair amount*, *resource accessibility*, *resource gaps*, and *crisis-specific services*.

Most participants reported that there are a fair number of community-based services available to service users, such as detox, addiction programs, walk-in clinics, other crisis services, and care coordination services [e.g., situation table (i.e., a network of service providers that use a case conference model to provide targeted care connections to individuals at imminent risk of crisis)]. Yet, participants reported issues regarding the accessibility and capacity of those resources.

In terms of *resource accessibility*, respondents described how a lack of alignment between community-based services’ operating hours (e.g., 9 am – 5 pm, Monday to Friday) and the CRT (24/7) hindered the ability to make “live” referrals (i.e., warm handoffs) or access the services when needed, therefore limiting their ability to divert individuals from the hospital or jail and make successful referrals when other services were closed (Quotes #3 and #4). They also reported that referral resources often lacked the staff or funding required to operate at full capacity, rendering them unable to accept referrals when needed. For example, eight participants (*n* = 3 leadership; *n* = 5 frontline) noted the medical detox facility was often at capacity and not accepting referrals (Quote #5). Lengthy waitlists at other services were identified as barriers to connecting CRT users to care and as a potential catalyst for crisis (Quote #6). Other present but insufficient services that were noted as needed to help address the root causes of crises were low-barrier detox facilities, shelters, crisis beds, primary care providers, residential treatment programs, counselling, and long-term services.

For the subtheme of *resource gaps***,** participants also identified areas in which services did not exist such as crisis facilities, mental health and addictions caseworkers, youth safe-sobering sites, and shelters specifically for people experiencing mental health and addictions.

Regarding *crisis-specific services*, participants identified the crisis service network as critical for CRT operation including the crisis phone and text line, the civilian-led mobile response team (mental health providers only), and crisis beds. Respondents reported that multiple levels of crisis services enabled the CRT to respond to higher acuity calls involving safety and welfare concerns (Quote #7), optimizing the use of police.

### Structure

The following section details CRT program resources and their perceived sufficiency. Four themes emerged within this area: *training*, *equipment/uniform*, *space*, and* staffing.*

#### Training

CRT frontline staff completed 40-h crisis intervention training (CIT). Document review of the CIT curriculum revealed that this training included information on the Mental Health Act of Ontario (MHA; RSO 1990, c. M.7; i.e., the legal requirement that dictates police must apprehend individuals they believe are to be of harm to themselves or others to be seen by a physician), specific disorders, and trauma and culturally informed care, and used simulated patients and pre-and-post testing. All police and mental health agency staff described the training as supplemental to past education and work experience (Quote #8). Crisis workers also received general training through the mental health agency, completed shadow shifts, and received situational awareness training through the police agency for safety.

#### Equipment/Uniform

Except for vehicles and radios which were provided by police, staff equipment was resourced by their respective employing organizations. Overall, participants from the police and mental health agency reported that the equipment was sufficient.

All crisis workers described their uniform, including its suitability for extreme temperatures, as essential for their safety and duties. The crisis workers also described their one-time footwear voucher as insufficient because they needed different pairs of footwear for different seasons. Participants from both agencies noted clothing allowances were lower for crisis workers than police and that this negatively impacted their working conditions and function (Quote #9).

#### Space

Participants reported that the crisis workers’ office was located within the police station (co-location) but had moved several times due to limited space within the police station.

#### Staffing

Staffing for each position was funded by their respective organization, except for a second crisis worker shift, funded through a grant from the provincial government. Respondents described issues with *staffing shortages* and *staff fit* as affecting the CRT.

Regarding *staffing shortages,* participants reported issues related to the number of crisis workers. Similarly, participants described occasional issues with having CRT officers available but also described efforts to mitigate this by spreading CIT training opportunities across platoons.

For the subtheme of *staff fit*, participants noted the importance of a “good fit” for CRT police officers and crisis workers. Fit was described in terms of work experience and personality or character traits (Quote #10). Participants noted that the crisis worker role may be difficult for new graduates, and educational experience does not guarantee that an individual will succeed in the position. In terms of police in the CRT role, participants noted that officers who were known as compassionate and interested in mental health tended to enjoy the CRT role best and were perceived to have the best performance. All police officers are self-elected to train for the CRT role.

### Process

CRT processes included the *dispatch process*, *call resolution*, and* collaboration.*

#### The Dispatch Process

Overall, the dispatch process was reported to be adequate. However, two subthemes emerged that hindered the dispatch process: *Responding to non-mental health calls* and *call volume and triage*.

All police officers and crisis workers described frustration at occasionally being *dispatched to non-mental health calls* because it interfered with their ability to respond to mental health calls when such calls came in (Quote #11). Participants explained that this was due to shortages in the availability of police officers to respond to community safety calls. Police officers and crisis workers noted that when they were on calls without a mental health component, the crisis worker was unable to do their intended role. However, participants also reported that this issue was mitigated when crisis workers could offer phone support to other officers who did not have a mental health worker in their car.

Related to the subtheme of *call volume and triage*, frontline and leadership staff from mental health and police agencies noted that there was high variability in mental health call volumes across different times and days, making it hard to anticipate when the call volume would increase. Participants also reported that when call volumes were high, many calls remained “pending” (i.e., in the response cue), resulting in the CRT being triaged to the highest priority call (Quote #12).

#### Call Resolution

Three sub-themes emerged related to the call resolution process.

The first and most prominent subtheme was *diversion*, *crisis de-escalation*, *and referrals*. Participants consistently shared that the main goal of the CRT was to divert individuals from the hospital and jail when appropriate. Participants from each agency shared that the mere presence of the crisis worker contributed to crisis de-escalation. All mental health agency staff mentioned that the crisis office was important for facilitating referrals, including access to a community resource list, but that service accessibility was a barrier to the referral process in general, including unclear referral processes.

The next subtheme was *hospital transfers*. Hospital wait times, described as typically several hours long, were identified as a barrier to timely call resolution by all participants. Participants from all agencies described wait times as burdensome, preventing CRT response to other calls.

Participants identified two primary issues related to long hospital wait times. First, the CRT waited in the ambulance bay, which participants described as loud, chaotic, extremely cold in the winter, and distressing for service users. During one of the ride-along visits, staff presented to the hospital for patient transfer. The service user was required to wait on a stretcher in the ambulance bay without privacy and among five paramedics and three police teams for more than 44 min, at which time the researcher was instructed to leave with a separate officer. There was no indication that the service user was to be assessed by a physician or nurse soon after the researcher left. Crisis and police staff confirmed that this was a typical occurrence.

Next, participants shared that service users’ presentations may change during long wait times, meaning that they may not appear apprehendable under the MHA by the time they are assessed by a physician and are thus sent home, potentially without appropriate care (Quote #13). Participants reported that this contributed to negative healthcare system interactions which may prevent future help-seeking and limit opportunities to address root issues (i.e., mental health or addiction issues).

Lastly, the subtheme of *nowhere else to go* emerged. Respondents often reported that although it was not always the appropriate service, service users often had nowhere to go other than hospital and/or jail, and resource gaps or resource accessibility issues seriously hindered their ability to divert individuals from these places (Quotes #14 and #15). During the ride-along site visits, a researcher observed an instance where this occurred (i.e., staff noted that hospital was likely not the best fit for the service user, but it was the only service option available). Several participants suggested that the presence of physical crisis facilities (e.g., respite beds) might mitigate this, since the existing crisis bed program was difficult to access due to stringent eligibility criteria dictated by the terms of a government grant.

### Outcomes

Respondents’ discussion of program outcomes and other qualitative data fit within three themes: *Client outcomes*, *quality of care*, and *program efficiencies*.

#### Client Outcomes

Participants most frequently reported hospital diversion and finding the most appropriate level of care as the intended outcomes of the program. Program documents, including annual reports, suggested that the program is achieving hospital diversions and improving in its capacity to do so (i.e., 55% of calls were classified as hospital diversions in the second year of operation, which was an 8% increase from the first year). In contrast, participants also identified that resource gaps and limited resource accessibility hinder the program’s ability for maximal hospital diversions (see ‘Complementary Community Resources’).

As supported by interview data and program document review, client dispositions were recorded as one of: care of self (i.e., alone, with family, or with service staff), hospital transfer by MHA apprehension or voluntary, detox, jail, emergency medical services, crisis beds, or shelter. All crisis workers shared the need to be resourceful to find alternative solutions to the hospital (Quote #16). Staff shared, however, that it was difficult to obtain follow-up information indicating if individuals connected with the services that they were referred to.

#### Quality of Care

When asked about the quality of the care provided by the team, responses were largely positive. Of note, some frontline staff shared that quality of care can be dependent on the amount of time they have with the service user, the presentation and cooperativeness of the service user, and the resources available to them at the time the interaction occurs. One police officer described their work as “[doing] the best [they] can with what [they’ve] got”.

Mental health agency leadership described monitoring care quality by reading post-contact notes. Others described receiving messages from service users and community organizations describing positive experiences. One hospital employee shared their experiences indicative of high-quality care from patient and family members interactions (Quote #17).

#### Program Efficiencies

Participants identified three main areas of cost savings and other efficiencies created by CRT pertaining to police, hospital, and other savings. Eleven participants identified savings for the police agency from reduced officer time on mental health calls as mental health workers were replacing officers onsite at calls that previously required additional police personnel. Participants also identified savings at the hospital resulting from ED diversions and reduced admissions (Quote #18).

Participants identified other perceived resource-savings to the broader health system including reduced EMS calls, increased community-based care, reduced repeat callers, and possible prevention of future justice system interactions. However, many of these suggested resource-savings were subjective and not testable by the authors through the data provided for this study. One police leadership person reported difficulties measuring resource savings that cut across more than one agency type. This suggests the need for CRT programs to include multi-agency data collection and analytics as part of their implementation plans and budgeting, especially during piloting when data is necessary for securing sustaining program funds.

## Discussion

### Summary of Findings

The purpose of this study was to describe the operations of a CRT in a geographically isolated, northern mid-sized city in Ontario, Canada, and extend learnings for similar communities also implementing a CRT. To do this, we described the CRT’s context, structure, process, and outcomes via interviews with leadership and frontline staff from the program and hospital, reviewed a range of program documents, and conducted ride-along site visits. Overall, the results suggest a good fit between the CRT model and the geographically isolated, northern mid-sized city context.

#### Context

Data from this study suggested that the northern context, including geography, temperature, workforce, and police mandate, and the different complementary community resources, including their accessibility and gaps in the resources available, affected the implementation and operation of the CRT. Like Thompson,^9^ our findings suggest that a high volume of mental health calls and a lack of community-based services create a need for a CRT program. The results of this study are also similar to Koziarski et al.,^16^ who identified a lack of community-based services to receive CRT referrals as a barrier to fulsome operation among several Canadian CRTs. These findings highlight the need to examine the community context, including mental health call volume and wrap-around community resource availability, when identifying the optimal crisis services model for a given community.^27^

#### Resources

Results from this study suggest that the program is sufficiently resourced in some areas, but not in others. Issues with staffing numbers and fit, as well as complementary community resources were prominent throughout this study. Challenges with funding and staffing CRTs are well-documented.^8,9,16,28^ and were expected in the current community where provincial skilled workforce shortages are compounded by a variety of unique or heightened health and social issues, resulting in poorer outcomes for individuals in these regions.^29^ Current efforts to alleviate the skilled health workforce shortage have been largely targeted at new graduates, such as the Ontario Learn and Stay Grant, in which recent healthcare graduates are incentivized to live and work in the North.^30^ While these approaches have yet to be applied to social service providers, our findings suggest these approaches may not be suitable for CRTs as study results and the general literature suggest that this role is better suited for those with more experience.^23,31^ Communities with workforce shortages in the areas of health and social services might anticipate how this may affect their program and how they operationalize it, including limiting hours, ongoing succession planning, and more frequent training plans or increased training budgets in case of high staff turnover.

#### Process

In general, qualitative findings suggested that the CRT is functioning well while also illuminating process challenges that were not captured in previous quantitative evaluations of this team. For instance, attending non-mental health calls was a barrier in the dispatch process. CRT dispatch to non-mental health calls is not well-documented in the literature. This is unlikely a unique challenge to this CRT and more likely a challenge in many rural or remote areas where police forces are generally short-staffed.^14^ Participants gave suggestions for how to mitigate resource waste by reallocating crisis worker time when this happens (e.g., phone support), triaging, and prioritizing mental health calls. Programs can monitor CRT non-mental health call response rates to ensure efficient and effective use of mental health resources.

Data also showed that the call resolution process worked well when the crisis could be de-escalated and a safety plan was formed. However, this process was challenged by long wait times at the hospital, a lack of suitable community-based services to receive persons diverted from hospital, and issues with connecting people to community services due to misaligned  operating hours, capacity limits, and/or, communication gaps. In interviews with officers from CRTs across Canada, participants also reported having no suitable place to bring individuals who do not need to be apprehended under the MHA and accessibility issues with the existing community-based services.^16^ Others^28^ similarly identified issues in successfully resolving calls due to resource availability. Issues regarding long wait lists, underfunding of community-based services, and the over-reliance on EDs for mental health care in Canada are well-described.^32^

The long wait times at the hospital were a large barrier for the present CRT. This is a common challenge for both CRTs and traditional police response teams^16,33–35^ and a uniquely circular issue for CRTs: Although CRTs potentially reduce wait times at the hospital by diverting individuals,^8,13,36–38^ they continue to be subject to long wait times when they are there which limits their ability to divert additional individuals through community response. An example of a possible solution to this issue comes from the police agency in Hamilton, ON, which has a Memorandum of Understanding (MOU) with the hospital stipulating that individuals who are brought in by police under the MHA are to be seen quickly to facilitate quicker departure from hospital for police.^39^ This agreement is attributed to having reduced wait times from around 150 to 70 min for traditional police responders and under an hour for the co-response team.^31^ However, this may be complicated for smaller and over-burdened hospitals that already struggle with meeting service prioritization demands or cities that only have one main hospital, as was the case for this CRT.

#### Outcomes

Overall, the qualitative findings of this study tentatively suggest that the CRT is achieving its aims for client outcomes, quality of care, and program efficiencies. The client outcome-related findings are aligned with scoping reviews and meta-analyses conducted on CRT (e.g., Marcus and Stergiopoulos; Ghelani et al.; Puntis et al.^12,13,40^). However, studies that include service user perspectives, data from several data sources (e.g., police and hospital data), and rigorous designs (e.g., comparison data, quasi-experimental, longitudinal) are needed to objectively assess outcomes.

Staff (leadership and frontline) perceptions of the quality of care given were largely positive. However, there were no formal client feedback mechanisms in this study allowing for consistent and unbiased opportunities. Structured (e.g., Ontario Perceptions of Care Tool;^41^ and unstructured (e.g., comment boxes) feedback channels can allow clients to comment on the quality of care and identify areas for improvement. For example, client feedback in studies by Evangelista et al.^42^ and Kirst et al.^35^ showed client preference for the CRT model compared to traditional response but identified a need for stronger referral pathways for long-term treatment options.

Overall, the program was perceived to be resource-saving and more efficient compared to traditional police response models. Participants identified savings separately for police, hospital, systems, and the community. One unpublished quantitative analysis of this program showed a direct savings of $574,000–$674,000 to the ED from hospital diversions alone in the team’s first two years of operation, not accounting for savings related to staffing or other avoided admissions. Data show that cost savings can come from officer time spent on calls, hospital transport costs, EMS calls, and greater systems costs through reduced repeat callers ^4,11^ and are consistent with other CRT research. ^15,43^ Data from other crisis system approaches further show that savings can be widespread throughout community service systems.^21^

### Building Crisis Systems

The results of this study support the emergent literature on crisis systems and system coordination.^11,21,22^ The presence of the crisis phone and text lines, a civilian-led mobile response team, and crisis beds were highly supportive of the CRT and created a stepped approach to addressing the crisis continuum. The Substance Abuse and Mental Health Services Administration’s^6^ best practice guidelines for behavioral health crisis care detail three core elements of a crisis system: (1) someone to talk to (i.e., crisis lines); (2) someone to respond (i.e., mobile crisis teams); (3) somewhere to go (i.e., crisis receiving and stabilization services).

In this study context, the two first elements of the crisis system were present. However, data from this study showed that there were gaps in the places to go. While there were crisis services, limited hours, limited capacity, or high-barrier eligibility criteria prevented individuals from being able to access these services. As a result, many individuals were required to present to the hospital, jail, or stay with family, when these were not the most appropriate options. A dedicated crisis facility may help to bridge gaps in the current crisis system and ease the burden of crises on the hospital and jail. Moreover, to fully complement the existing crisis system, the crisis facility must have a 24/7 availability, a “no wrong door” policy, and rapid drop-off for police or paramedics.^21^ However, such facilities will inevitably be affected by the community-wide staffing and funding shortages, and are less frequent in rural, isolated, or smaller communities.^14^ Even without the full scope of system services, data from this study show that smaller or remote cities can still benefit from a CRT model if other aspects of a crisis system are established (e.g., crisis lines and civilian-led mobile crisis response).

The U.S.-based CrisisNow.com website has a Crisis Resource Need Calculator, which can allow communities to understand the potential healthcare costs associated with crisis care delivery across a variety of crisis system scenarios, including a traditional healthcare system, CRTs, and a comprehensive crisis system including crisis centres.^44^ Such accessible tools to calculate possible costs in Canada may assist with justification for the employment of CRTs or other crisis response models. Based on extant research, we expect that such a calculator might show that a CRT model may not be cost-effective initially, especially in smaller or rural communities ^7,45^ because it is high-cost and best suited for high-acuity cases.^23,46^ Calculators that can balance complementary crisis approaches may be needed to optimize service and efficiency in smaller communities with fewer levels of crisis support.

Fix et al.^7^ state that “while there are best practices, there may not be one ‘right’ way to do things; each community or region should evaluate their resources and options, and work to design a [crisis] system that ensures [people in mental health and addictions crisis] have safe and appropriate levels of care offered” (p. 208). Therefore, communities must holistically examine their existing services and take a systems approach to addressing mental health and addiction crises.

### Limitations

This is a case study conducted in a remote northern city in Canada, which may limit the generalizability of findings (i.e., may not be applicable to larger communities or different countries that operate under different laws and systems). Case studies can identify practices that work or do not work in a particular context, which may inform these practices in other contexts (i.e., different communities with differing needs).^47^ At the same time, despite context variations, CRT programs frequently experience similar barriers, such as funding and staffing. With these findings, general assumptions may be made about the adaptability of the CRT model to meet the needs of other contexts and relevant considerations. Future research should continue to examine the CRT model in contexts that vary from the typical large urban centres. Moreover, future research that uses quantitative outcomes and quasi-experimental or longitudinal designs is needed to rigorously assess CRT outcomes. Indeed, a lack of quantitative data to demonstrate resource savings across the broader healthcare system (i.e., beyond the police agency and hospital) limited our ability to support the information provided by participants.

Additional limitations to this study include the small sample size. Due to the small population size and richness of interviews (*M* = 55 min in length), however, we believe this sample size is appropriate for this study. To avoid homogeneity of sampling and to ensure various experiences are captured in interviews, we purposively sampled crisis workers and police officers from multiple platoons and shifts and varying time spent working with the team. We did not have information to understand any differences between those who volunteered for the study and the overall workforce population who were eligible to participate. Additionally, we did not calculate intercoder reliability in the thematic analysis.

The lack of service user perspectives is an important limitation of this research. Some past research has included qualitative accounts of service users’ experience (e.g., Lamanna et al.; Kirst et al.^8,35^) and future research should continue to involve service users. Furthermore, we did not interview 911 dispatchers, who may have provided key insights into the triage process and call volumes. While this is the first study, to our knowledge, to interview hospital staff regarding CRTs, future studies should continue to seek out diverse perspectives from stakeholders and key players.

## Implications for Behavioral Health

The present study demonstrates how the CRT model is applied to a remote northern context. The results support the importance of identifying community needs, choosing an aligned model for their community (i.e., tailored for community needs and resources), and forming strong partnerships across agencies. It provides specific program components and processes that, given a particular context, may be helpful for planning and making the program work.

This work further emphasizes the need to prioritize creating a cohesive crisis system with various service options tailored across the crisis continuum to assist with CRT call prioritization. It shows how communities can make intentional and informed decisions regarding where to expand crisis services based on the existing options. This study has the potential to inform other communities as to how CRTs can be implemented in a range of contexts or improve current operations.

For communities that currently have a CRT, data show aspects of program evaluation and progress monitoring that can improve the impact and efficiency of the program and identify possible gaps in service.^23^ This, in turn, can inform decisions for CRT operations and service creation prioritization within the community. Partnering with universities or colleges can assist with program evaluation as there is often mutual benefit (i.e,. knowledge pursuit and research experience for students) and agencies may not have staff with expertise in program evaluation or may wish for program evaluation to be conducted externally. Community safety and well-being plans (e.g., see^48^) are one option for identifying and organizing collective efforts to address mental health and addictions across different agencies within a community, including police agencies.

## Supplementary Information

Below is the link to the electronic supplementary material.

Supplementary file1 (DOCX 18.7 KB)

Supplementary file2 (DOCX 17.9 KB)

Supplementary file3 (DOCX 15.1 KB)

Supplementary file4 (DOCX 16.1 KB)

## Data Availability

The participants of this study did not give written consent for their data to be shared publicly, so due to the sensitive nature of the research, supporting data is not available.
