# Supplementary material for: Matching Mobile Crisis Models to Communities: An Example from Northwestern Ontario
Source: J Behav Health Serv Res. 2024 Apr 30;51(3):355–76. doi: 10.1007/s11414-024-09882-7 (PMC11180628; doi:10.1007/s11414-024-09882-7)
Supplement: Supplementary file 2 — Supplementary file2 (DOCX 17.9 KB) [file 11414_2024_9882_MOESM2_ESM.docx]

Supplementary File 2

Crisis Worker Interview Guide

**General Questions**

1. Tell us about your role in the [CRT] program.
2. What formal education do you have?
3. What specific training did you receive for the [CRT] program?
4. How long have you been working with [the CRT]?
5. What are your general impressions about the [CRT] program?
   1. Can you think of any primary benefits?
   2. What about challenges of this program?

**Structure**

1. Now, I would like to ask you about program resources. I will be mentioning different component and would like to hear about your opinions on how the program is resourced for each component?
2. Staff
3. Staff training and credentials… do you think that the training your received is appropriate for your position? What about other staff members? Is training consistent?
4. Necessary equipment, these are things such as your own desk and chair, computers, phones, appropriate safety equipment, uniforms, or vest ID badges?
5. Are you able to access private spaces if needed?
6. Funding
7. Are there any other resources that you can think of?
8. Overall, how do you think the resources of the program support or prevent the [CRT] program from achieving its goals?
9. Next, I would like to ask you about community resources. How do you think the community resources in [city name] complement the [CRT] program which relies on referrals to low-barrier community care? **[Context]**

**Process**

For the next few questions, I will be asking you about the functioning of the [CRT] team. When you think about these, I want you to think about the process of this program rather than outcomes. So, these are the things that help or hinder achieving the goals of the [CRT] program.

1. How do you find the overall dispatch process?
   1. Can you think of things that help you to do your job or make it harder?
2. What helps you to successfully give community resource referrals when needed? ]
3. Are there any barriers in this process?
4. Are there any barriers to successfully transferring individuals to the hospital when needed?
5. Anything that helps this process?
6. How often is [the CRT] unable to answer calls for service?
7. What happens when [the CRT] is not available?
8. How do you collaborate with the [CRT] worker to do your job?
9. How about the hospital staff?
10. Overall, what makes a call run smoothly?
11. What are common challenges that you haven’t mentioned?
12. Can you think of any policies or procedures are facilitating or hindering the functioning of this program?
    1. Are there any policies or procedures that you would like to see developed?

**Outcome**

Next, I will be asking you about the outcomes of the [CRT] program.

1. How is the program achieving intended client outcomes?
   1. What might make this easier or prevent this from happening?
2. What do you think about the quality of care provided by the [CRT] team?
   1. How does the [CRT] program consider clients’ individual needs?
3. What is your overall perceptions on the resource saving of the [CRT] program compared to traditional police response? [Prompt: police resources; hospital resources]
4. What is your overall experience working on the [CRT] team?
   1. Are you proud or satisfied with the service you provide?
   2. Can you provide any insight on your overall job sustainability or burnout in this role?
5. To what extent do you believe the program provides high-quality and appropriate response to mental health calls?

**Context**

1. Lastly, the [CRT] model was designed and tested for large urban centres, do you believe that the [CRT] model is a good fit for [city name]? Why or why not?

Prompts:

- 1. Community demographics?
  2. Geography?
  3. Police workforce size?
  4. Community resources?

1. Do you have any recommendations for [CRT] program or changes that you would like to see?

Is there anything else you would like to share?
