# Supplementary material for: Matching Mobile Crisis Models to Communities: An Example from Northwestern Ontario
Source: J Behav Health Serv Res. 2024 Apr 30;51(3):355–76. doi: 10.1007/s11414-024-09882-7 (PMC11180628; doi:10.1007/s11414-024-09882-7)
Supplement: Supplementary file 3 — Supplementary file3 (DOCX 15.1 KB) [file 11414_2024_9882_MOESM3_ESM.docx]

Supplementary File 3

Hospital Staff and Leadership Interview Guide

**Questions**

1. What is your role at [the hospital]?
2. What formal education do you have (e.g., nursing degree, physician etc.)?
3. What do you know about the [CRT] program, including its purpose and goals?
4. What is your understanding of how the [CRT] program is supposed to interface with the hospital?
5. Are there any policies or procedures that you can think of that facilitate or hinder the functioning of the [CRT] program?
6. To what extent is the hospital is adequately equipped to support the [CRT] program and its goals?
7. Are there any barriers or facilitators to assessing or admitting individuals who come to the emergency department via the [CRT] program?
   1. Can you think of any way to smooth this process?
8. Do you think the community of [city name] is a good fit with the [CRT] model? Why or why not?
   1. How do you think the community resources in [city name] complement the [CRT] program which relies on referrals to low-barrier community care?
9. What other information would you like to share with us about your views on the value and function of the [CRT] program at [the hospital] and in [city name]?
10. Do you have any recommendations for the [CRT] program or changes you would like to see?

Is there anything else you would like to share?
