# Supplementary material for: Matching Mobile Crisis Models to Communities: An Example from Northwestern Ontario
Source: J Behav Health Serv Res. 2024 Apr 30;51(3):355–76. doi: 10.1007/s11414-024-09882-7 (PMC11180628; doi:10.1007/s11414-024-09882-7)
Supplement: Supplementary file 4 — Supplementary file4 (DOCX 16.1 KB) [file 11414_2024_9882_MOESM4_ESM.docx]

Supplementary File 4

Mental Health and Police Agency Leadership Interview Guide

**General Questions**

1. Tell us about your role at [organization].
   1. What hours/days do you work?
2. What formal education do you have?
3. How are you involved with the [CRT] program? Did you receive any specific training for this?
4. How long have you been working with [the CRT]?
5. What are your general impressions about the [CRT] program?
   1. Can you think of any primary benefits?
   2. What about challenges of this program?

**Structure**

1. Now, I would like to ask you about program resources. I will be mentioning different component and would like to hear about your opinions on how the program is resourced for each component?
2. Staff
3. Staff training and credentials… do you think that the training your staff received is appropriate for the position? Is training consistent?
4. Necessary equipment, these are things such as your own desk and chair, computers, phones, appropriate safety equipment, uniforms, or vest ID badge for the staff? For yourself?
5. Funding
6. Are there any other resources that you can think of?
7. Overall, how do you think the resources of the program support or prevent the [CRT] program from achieving its goals?
8. Next, I would like to ask you about community resources. How do you think the community resources in [city name] complement the [CRT] program that relies on referrals to low-barrier community care? **[Context]**

**Process**

For the next few questions, I will be asking you about the functioning of the [CRT] team. When you think about these, I want you to think about the process of this program rather than outcomes. So, these are the things that help or hinder achieving the goals of the [CRT] program.

1. How do you find the overall dispatch process?
   1. Can you think of things that facilitate or hinder this process?
2. Can you think of any barriers or facilitators for [CRT] staff when giving community resource referrals?
   1. How does the program collaborate with community organizations to provide these referrals?
   2. Is the cultural appropriateness of these resources taken into account?
3. Are there any barriers to successfully transferring individuals to the hospital when needed?
   1. Anything that helps this process?
   2. How does the program collaborate with the hospital to ease this process?
4. How often is [CRT] unable to answer calls for service?
   1. What happens when [CRT] is not available?
5. How do partner agencies collaborate to implement this program?
   1. What about community agencies?
6. Can you think of any policies or procedures that are facilitating or hindering the functioning of this program?
   1. Are there any policies or procedures that you would like to see developed?
7. Can you think of anything else that either helps or hinders how the program is run?

**Outcome**

Next, I will be asking you about the outcomes of the [CRT] program.

1. How is the program achieving intended client outcomes?
   1. What might make this easier or prevent this from happening?
2. What do you think about the quality of care provided by the [CRT] team?
   1. How does the [CRT] program consider clients’ individual needs?
3. What is your overall perceptions on the resource saving of the [CRT] program compared to traditional police response? [Prompt: police resources; hospital resources]
4. What is your perception of [CRT] frontline workers’ experiences?
   1. Can you provide any insight on satisfaction with service?
   2. Can you provide any insight on employee job sustainability or burnout? Is there high job turnover in this program?

**Context**

1. Lastly, the [CRT] model was designed and tested for large urban centres, do you believe that the [CRT] model is a good fit for [city name]? Why or why not?

Prompts:

1. Community demographics?
2. Geography?
3. Police workforce size?
4. Community resources?
5. Do you have any recommendations for [CRT] program or changes that you would like to see?

Is there anything else you would like to share?
